# DNA methylation markers have universal prognostic value for anal cancer risk in HIV‐negative and HIV‐positive individuals

**DOI:** 10.1002/1878-0261.12926

**Published:** 2021-03-16

**Authors:** Ramon P. van der Zee, Carel J.M. van Noesel, Ivonne Martin, Timo J. ter Braak, Daniëlle A.M. Heideman, Henry J.C. de Vries, Jan M. Prins, Renske D.M. Steenbergen

**Affiliations:** ^1^ Department of Pathology Cancer Center Amsterdam Amsterdam UMC Vrije Universiteit Amsterdam Amsterdam The Netherlands; ^2^ Department of Internal Medicine Division of Infectious Diseases Amsterdam Institute for Infection and Immunity Amsterdam UMC University of Amsterdam Amsterdam The Netherlands; ^3^ Department of Dermatology Amsterdam UMC University of Amsterdam Amsterdam The Netherlands; ^4^ Department of Pathology Cancer Center Amsterdam Amsterdam UMC University of Amsterdam Amsterdam The Netherlands; ^5^ Department of Epidemiology and Data Science Amsterdam UMC Vrije Universiteit Amsterdam Amsterdam The Netherlands; ^6^ STI Outpatient Clinic Department of Infectious Diseases Public Health Service of Amsterdam (GGD Amsterdam) Amsterdam The Netherlands

**Keywords:** anal cancer, anal intraepithelial neoplasia, host cell DNA methylation markers, HIV, human papillomavirus

## Abstract

Anal cancer has increasing incidence and is preceded by high‐grade anal intraepithelial neoplasia (HGAIN; AIN2–3). Previously, we identified and validated several methylation markers for accurate detection of anal cancer and HGAIN with cancer risk in HIV‐positive (HIV+) men who have sex with men (MSM). This study aimed to evaluate these markers in HIV‐negative risk groups. A cross‐sectional series of 176 tissue samples of anal cancer, AIN3, AIN2, AIN1 and control biopsies obtained in HIV‐negative women and men was tested for six methylation markers (*ASCL1, LHX8, SST, WDR17, ZIC1* and *ZNF582*). Accuracy for detection of AIN3 and cancer (AIN3+) was determined by univariable and multivariable mixed‐effect ordinal logistic regression. Methylation levels of all markers increased with increasing severity of disease (*P* < 0.0001) and were comparable to results in HIV+ MSM. All markers showed high accuracy for AIN3+ detection [area under the curve (AUC): 0.83–0.86]. The optimal marker panel (*ASCL1* and *ZIC1*; AUC = 0.85 for AIN3+) detected 98% of cancers at 79% specificity. In conclusion, DNA methylation markers show a high diagnostic performance for AIN3+ detection in HIV+ and HIV‐negative risk groups, justifying broad application of methylation analysis for anal cancer prevention programmes.

## Graphical abstract

Host cell DNA methylation plays a role in anal carcinogenesis in HIV‐negative (HIV‐neg) and HIV‐positive (HIV+) individuals, including men who have sex with men (MSM). Methylation levels are increased in high‐grade anal intraepithelial neoplasia at risk of progression towards anal cancer, making methylation markers promising prognostic biomarkers.

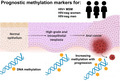

**Abbreviations**
*ACTB*
: β‐actin
AIN: anal intraepithelial neoplasia
AUC: area under the curve
CI: confidence interval
CpG: cytosine located 5′ of a guanine
Cq: quantification cycle
EIA: enzyme immunoassay
FFPE: formalin‐fixed paraffin‐embedded
HGAIN: high‐grade anal intraepithelial neoplasia
HPV: human papillomavirus
HRA: high‐resolution anoscopy
hrHPV: high‐risk human papillomavirus
IBD: inflammatory bowel disease
IQR: interquartile range
lrHPV: low‐risk human papillomavirus
*J*
: Youden's Index
LOOCV: leave-one-out cross-validation
lrHPV: low‐risk human papillomavirus
MSM: men who have sex with men
non‐CV: non‐cross‐ validated
ns: nonsignificant
PP: predicted probabilities
qMSP: quantitative methylation‐specific PCR
ROC: receiver operating characteristics
SOTR: solid organ transplantation
SCC: squamous cell carcinoma
WTS: whole tissue section

## 1 Introduction

Anal cancer incidence is increasing worldwide. Although still relatively rare in the general population (age‐adjusted incidence rates of 1–2 per 100 000 person‐years), incidence rates are slightly higher (1.5–2‐fold) in women compared with men, increase with increasing age and are disproportionally high in several high‐risk groups [1, 2]. HIV‐positive (HIV+) men who have sex with men (MSM) have the highest incidence rates (85 per 100 000), followed by HIV+ men who have sex with women (32 per 100 000), and HIV+ women (22 per 100 000) [3]. Besides the role of HIV, iatrogenic systemic immunosuppression, for example, for solid organ transplantation or autoimmune diseases (including inflammatory bowel diseases (IBDs), in particular Crohn's disease, or haematological malignancies) also increases the risk for anal cancer (up to 12 per 100 000). HIV‐negative MSM (19 per 100 000) and women with prior human papillomavirus (HPV)‐induced (pre)cancer also have substantially higher incidence rates (cervical: 6–9 per 100 000 and vulvar: 42–48 per 100 000) [3].

Almost all anal cancers are squamous cell carcinoma (SCC) and are, similar to cervical cancer, mainly caused by a persistent high‐risk (hr)HPV infection and preceded by precursor lesions: anal intraepithelial neoplasia (AIN; graded 1–3) [4]. High‐grade AIN (HGAIN; AIN2–3), also called anal high‐grade squamous intraepithelial lesions, can progress to cancer [5, 6]. HGAIN is highly prevalent in HIV+ MSM (29%) and in HIV‐negative MSM (22%), but prevalence data on other risk groups are scarce [6, 7].

In analogy with cervical cancer screening, screening and treatment of HGAIN to prevent anal cancer in high risk groups is under debate. High‐resolution anoscopy (HRA)‐guided biopsies, the gold standard for HGAIN diagnostics, is a burdensome, expensive and complicated procedure [7]. Alternative screening techniques, such as anal cytology and HPV testing, have suboptimal diagnostic performance [4, 7, 8].

Host cell DNA methylation, that is addition of methyl groups (hypermethylation) to cytosines in cytosine located 5′ of a guanine (CpG) sites, is an epigenetic hallmark in HPV‐induced carcinogenesis, which can lead to inactivation of tumour suppressor genes [9]. Recently, we established that host cell DNA methylation is associated with anal carcinogenesis in HIV+ men and identified several methylation markers for accurate detection [area under the curve (AUC = 0.90)] of HGAIN and anal cancer in HIV+ MSM [10]. Subsequently, we validated the most potent markers (*ASCL1, LHX8, SST, WDR17, ZIC1* and *ZNF582*) in a large independent series, confirmed the accuracy (AUC = 0.89) and showed that high methylation levels are associated with progression towards cancer. We therefore consider methylation analysis using these markers to be a promising tool to detect HGAIN and anal cancer in HIV+ MSM and for cancer risk stratification, identifying HGAIN in need of treatment, thereby reducing overtreatment [11].

In present study, we aimed to investigate whether host cell DNA methylation also plays a role in anal carcinogenesis in other risk groups, namely HIV‐negative women and men referred for anal complaints or in screening for being at risk for anal cancer, and evaluated the diagnostic potential of the earlier identified six methylation markers in the detection of HGAIN and anal cancer in these risk groups.

## 2 Materials and methods

### 2.1 Clinical specimens

This study involved a molecular analysis of a cross‐sectional series of formalin‐fixed paraffin‐embedded (FFPE) anal tissue samples across the full spectrum of anal (pre)cancer lesions in HIV‐negative women and men (Table 1). These patients were referred to the Amsterdam University Medical Centers (Amsterdam UMC), Location Academic Medical Center (AMC), Amsterdam, The Netherlands, for anal complaints or were in screening for having an increased risk (see risk factors below) of developing anal cancer. A total of 176 samples, including 40 anal SCC specimens (obtained between 1985 and 2011 in 40 patients: 22 women and 18 men) and 136 biopsies [normal control samples *n* = 30; AIN1 (including anogenital condylomata) *n* = 57; AIN2 *n* = 21; AIN3 *n* = 28; obtained between 2006 and 2020 in 81 patients: 35 women and 46 men], were retrieved from the Pathology Archive of the Amsterdam UMC. For 39 patients, multiple biopsies from different lesions were included. Normal control samples included biopsies taken from lesions suspected for AIN but histopathologically graded as normal or reactive anal epithelium. Median age of patients at time of biopsy per histological group and per gender is reported in Table 1. Of patients of whom biopsies were included, electronic medical records were thoroughly reviewed for HIV status and other risk factors for anal cancer (Table S1). HIV status (positive/negative) was tested or patient reported. For 18 anal SCC samples of older women, the HIV status was not provided in the medical record (no mentioning or test result) and was considered HIV‐negative (presumed HIV‐negative). Other reported risk factors we looked for were being MSM; earlier or concurrent cervical (pre)cancer or vulvar (pre)cancer; solid organ transplantation recipient (SOTR); receiving other systemic immunosuppression therapies; and IBD (Crohn's disease or ulcerative colitis).

**Table 1:** Number of included tissue samples of HIV‐negative men and women and age at biopsy per histological category. Data are *n*, numbers or median [interquartile range (IQR)]. Numbers represent samples. Consequently, patients with multiple samples can be represented in multiple categories. Normal: normal control samples; AIN: anal intraepithelial neoplasia (grades 1–3); SCC: anal squamous cell carcinoma.

Histological category	Total no. of samples	HIV‐negative men		HIV‐negative women	
		Subtotal	Age at biopsy, years	Subtotal	Age at biopsy, years
Normal	30	15	53 [46–69]	15	61 [46–67]
AIN1	57	38	44.5 [29–54]	19	49 [41–57]
AIN2	21	11	52 [45–60]	10	44 [39–58]
AIN3	28	9	63 [52–68]	19	52 [4–62]
SCC	40	18	56.5 [51–65]	22	58.5 [44–77]
Total	176	91	52 [41–62]	85	52 [44–65]

For comparison, we used previously published methylation data from a large cross‐sectional series of anal (pre)cancer tissue samples (*n* = 345; 30 anal SCC, 74 AIN3, 98 AIN2, 37 AIN1 and 106 normal) of HIV+ MSM tested with the same methylation markers and assays. This concerned a population in screening for anal (pre)cancer at the Amsterdam UMC. Further details have been reported previously [11].

### 2.2 Ethics

We adhered to the Code of Conduct for Responsible Use of Left‐over Material of the Dutch Federation of Biomedical Scientific Societies, and ethical approval for use of archived biopsies was waived by the Institutional Review Board of the Amsterdam UMC [ref. no. 17/151 (SCC) and 18/341 (AIN biopsies)].

### 2.3 Histopathological review

Whole tissue sections (WTS) of FFPE tissue blocks were sectioned according to the sandwich method, as described previously [11]. The first and last sections were haematoxylin‐ and eosin‐stained and histopathologically reviewed by a board‐certified pathologist (CJMvN), experienced in AIN histopathology, for confirmation of the lesion and to render a revision diagnosis for analysis. In‐between WTS were collected in sterile PCR tubes for DNA isolation. The highest grade of AIN represented in the sandwich of the study specimen was considered the diagnosis for this study. Whenever this review was inconclusive (cases difficult to classify), p16^INK4A^ immunohistochemistry was used to determine the final study diagnosis [12].

### 2.4 DNA isolation and HPV testing

DNA was isolated using the QIAamp DNA FFPE tissue kit (Qiagen, Hilden, Germany) according to the manufacturer's instructions and eluted with the easyMAG 3 elution buffer (bioMérieux, Boxtel, The Netherlands), as described before [11]. HPV testing and genotyping was performed on isolated DNA by conducting a general primer GP5+/6+‐mediated PCR with hybridisation of PCR products in an enzyme immunoassay (EIA). First, an oligonucleotide probe cocktail that detects 14 hrHPV types (HPV16, 18, 31, 33, 35, 39, 45, 51, 52, 56, 58, 59, 66 and 68) was used with subsequent genotyping of EIA‐positive samples using a microsphere bead‐based assay (Luminex xMAP; Luminex Corp, Austin, TX, USA). For samples negative in hrHPV EIA, another oligonucleotide probe cocktail was applied that detects low‐risk HPV (lrHPV) types (HPV6, 11, 26, 30, 32, 34, 40, 42, 43, 44, 53, 54, 55, 57, 61, 64, 66, 67, 69, 70, 71, 72, 73, 81, 82, 83, 84, 85, 86, 89 and 90), without further genotyping. Samples negative in both hrHPV EIA and lrHPV EIA were considered HPV‐negative after verification of human DNA using β‐globin amplification.

### 2.5 DNA methylation analysis using multiplex quantitative methylation‐specific PCR

For DNA methylation analysis, isolated DNA was bisulfite‐converted using the EZ DNA Methylation Kit (Zymo Research, Orange, CA, USA) according to the manufacturer's instructions, converting unmethylated cytosines into uracils while conserving methylated cytosines, as described before [11]. EpiTect MethyLight Master Mix (Qiagen) was used, together with fluorescent dye‐labelled probes, 50 ng input of bisulfite‐converted DNA and 100–300 nm of each primer, specifically amplifying methylated bisulfite‐converted DNA. H_2_O was used as negative control. Bisulfite‐converted sample DNA was analysed for six methylation markers (*ASCL1, LHX8, SST, WDR17, ZIC1* and *ZNF582*) using two multiplex quantitative methylation‐specific PCR (qMSP) assays, each targeting three genes and the reference gene, β‐actin (*ACTB*). One multiplex qMSP targeted *ASCL1, LHX8* and *ZNF582*, while the other assay targeted *SST, WDR17* and *ZIC1* [11]. A cycle threshold of < 32 for *ACTB* indicated sufficient DNA, DNA quality and adequate bisulfite conversion [11]. The multiplexes were performed on the ViiA 7 Real‐Time PCR System (Applied Biosystems, Foster City, CA, USA) using a double‐stranded DNA‐based calibrator as internal quality control, which contains the amplicon sequences of the targets and *ACTB* [13]. Cycle threshold values were measured at fixed thresholds for fluorescence. ΔΔCq ratios were computed using the comparative quantification cycle (Cq) method by comparing the target Cq values with the Cq values of *ACTB* and of the internal quality control calibrator (2^−ΔΔCq^ × 100) [14, 15].

### 2.6 Statistical analysis

Differences in methylation levels across the different histological categories (normal, AIN1–3, SCC) within and across risk groups (HIV‐negative women, HIV‐negative men, HIV+ MSM) and HPV status classes were visualised using boxplots and tested for statistical significance using the Kruskal–Wallis test, followed by pairwise Mann–Whitney *U*‐tests, with the Bonferroni correction.

For evaluation of the diagnostic performance of the markers, we performed univariable mixed‐effect ordinal logistic regression for the individual markers, while multivariable mixed‐effect ordinal logistic regression with stepwise backward selection based on the likelihood ratio criteria was used to obtain and evaluate an optimal marker panel [16, 17, 18]. We used three ordered classes of outcomes: [≤ AIN1] (normal control samples and AIN1), [AIN2] and [AIN3+] (AIN3 and anal SCC). To account for a correlation between multiple samples obtained from the same patient, we incorporated a random subject effect in the model. From the regression analyses, we obtained predicted probabilities (PP; values ranging from 0 to 1), representing the risk for each class outcome given the methylation marker level of a sample. In interpreting the PP, care should be taken that these probabilities are based on the distribution of the class labels in the study and not in the general population. For instance, a class that has fewer samples compared with the other might perform poor in predicting the true class.

Compared with the use of logistic regression analysis in our previous papers on HIV+ MSM, ordinal regression better accounts for the ordered nature of the data (normal to AIN1–3 to SCC). Moreover, the ordinal regression analysis enables the inclusion of the intermediate class of AIN2 samples in the prediction model [17, 18]. For practical reasons and clinical applicability, we converted the ordinal outcome in a binary outcome by collapsing the PP of the three classes into cases [AIN3+] (AIN3 and anal SCC) or controls [≤ AIN1] (normal control samples and AIN1) and dividing the PP of the middle class [AIN2] into half to contribute to the PP of cases and controls.

Diagnostic performance of the models was visualised using receiver operating characteristic (ROC) curves and assessed through the AUC, as well as through the sensitivity and specificity at the Youden's Index (*J*) threshold (threshold that maximises the sum of sensitivity and specificity). For samples with PP above the *J*‐threshold, methylation results were considered methylation‐positive. To evaluate the performance of our models on samples outside the set, we performed leave‐one‐out cross‐validation (LOOCV). All analyses were performed on log_2_‐transformed ΔΔCq ratios.

For comparison, we applied the optimal marker panel developed for AIN3+ detection in HIV+ MSM in previous study [11]. Therefore, the multivariable logistic regression model, combining markers *ZNF582, ASCL1* and *SST*, was used to compute PPs and determine methylation‐positive detection rates based on the non‐cross‐validated (non‐CV) *J*‐threshold (≥ 0.434) [11]. Due to differences in populations and model development, a further comparison in AUC, sensitivity and specificity was not possible.

Statistical analyses and data visualisation were performed using the r Statistical Software (version 3.6.1; Foundation for Statistical Computing, Vienna, Austria) with packages ordinal [19], buildmer [20], pROC [21], and ggplot2; IBM spss Statistics software (version 26; IBM Corporation, Armonk, NY, USA); and GraphPad Prism (version 8.2.1; graphpad Software Inc., La Jolla, CA, USA). Reported *P*‐values are two‐sided, with 0.05 as the significance threshold.

## 3 Results

### 3.1 HPV prevalence in anal tissue samples

Table 2 shows the HPV testing and hrHPV genotyping results in anal tissue specimens. Forty‐three per cent of normal control samples (13 of 30) was tested HPV‐positive, all lrHPV. HPV was detected in 95% (54 of 57) of AIN1, 95% (20 of 21) of AIN2 and all (28 of 28) AIN3. AIN1 was predominantly lrHPV‐positive, whereas AIN2 and AIN3 were predominantly hrHPV‐positive. Multiple hrHPV infections were found in 17% (one of six) of hrHPV‐positive AIN1, 19% (three of 16) of hrHPV‐positive AIN2 and 9% (two of 22) of AIN3. HPV was detected in 83% (33 of 40) of SCC, all single infections and predominantly hrHPV (94%; 31 of 33). Overall, HPV16 was the predominant hrHPV type, found in 60% of HPV‐positive AIN2, 68% of AIN3 and 85% of HPV‐positive SCC.

**Table 2:** HPV prevalence and HPV genotype distribution per histological category in WTS of anal samples. HPV risk classification based on International Agency for Research on Cancer (IARC) classification [29, 30]. Type‐specific positivity includes those contributed by multiple infections. One sample can therefore be counted more than once. Total number of tissue samples tested: 176 (including 28 HPV‐negative). Normal: normal control samples; AIN: anal intraepithelial neoplasia (grades 1–3); SCC: anal squamous cell carcinoma; WTS: whole tissue section.

HPV genotyping results per histological category					
HPV type	Normal (*n* = 30)	AIN1 (*n* = 57)	AIN2 (*n* = 21)	AIN3 (*n* = 28)	SCC (*n* = 40)
Positivity for any HPV type	13/30 (43%)	54/57 (95%)	20/21 (95%)	28/28 (100%)	33/40 (83%)
High‐risk HPV					
Positivity for any high‐risk HPV type	0/13 (0%)	6/54 (11%)	16/20 (80%)	22/28 (79%)	31/33 (94%)
HPV16		3/54 (6%)	12/20 (60%)	19/28 (68%)	28/33 (85%)
HPV18		1/54 (2%)	2/20 (10%)	1/28 (4%)	1/33 (30%)
HPV31				1/28 (4%)	
HPV33				4/28 (14%)	2/33 (6%)
HPV35			1/20 (5%)		
HPV39					
HPV45					
HPV51					
HPV52					
HPV56			2/20 (10%)	1/28 (4%)	
HPV58			1/20 (5%)		
HPV59		1/54 (2%)	2/20 (10%)		
HPV66^a^		1/54 (2%)			
HPV68^a^					
Multiple hrHPV infections in hrHPV‐positive in WTS^b^	0/0 (0%)	1/6 (17%)	3/16 (19%)	2/22 (9%)	0/31 (0%)
Low‐risk HPV					
Positivity for any lrHPV type^c^	13/13 (100%)	49/54 (91%)	8/20 (40%)	8/28 (29%)	2/33 (6%)

^a^ Gathered under hrHPV, although formally considered as probable/possible hrHPV [29, 30].

^b^ Only hrHPV (including probable/possible hrHPV) types taken into account.

^c^ lrHPV types in hrHPV‐neg samples taken into account.

### 3.2 Methylation levels across histological categories of anal disease

**Fig. 1:**
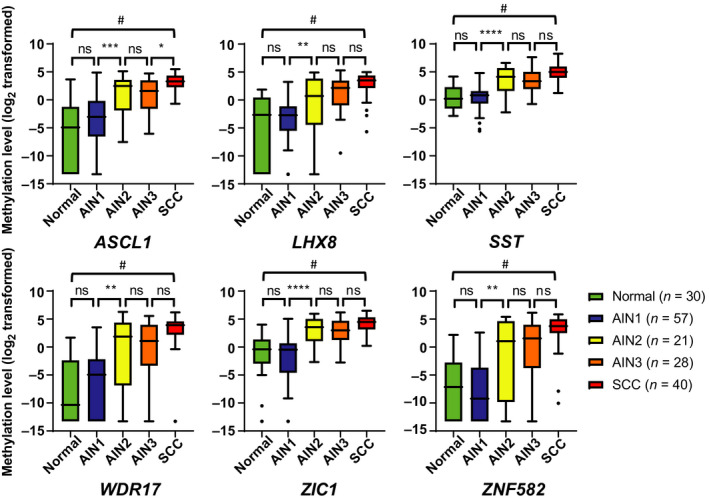
Methylation levels increase with severity of anal disease. Boxplots of DNA methylation levels relative to a reference gene *ACTB* (log_2_‐transformed ΔΔCq ratios; *y*‐axis) in the different histological categories of anal tissue samples of HIV‐negative women and men (*x*‐axis) for six markers: *ASCL1*, *LHX8*, *SST*, *WDR17*, *ZIC1* and *ZNF582*. The box of the boxplots bounds the IQR divided by the median, Tukey‐style whiskers extend to a maximum of 1.5 IQR beyond the box, and ● marks an outlier sample. Overall differences between histological categories upon Kruskal–Wallis omnibus test (^#^ *P* < 0.0001), followed by *post hoc* testing using the Mann–Whitney *U*‐test and Bonferroni multiple testing correction: *P < 0.05, **P < 0.01, ***P < 0.001, ****P < 0.0001, ns: nonsignificant.Normal, normal control samples; AIN: anal intraepithelial neoplasia (grades 1–3); SCC: anal squamous cell carcinoma.

Methylation levels of all six markers differed significantly (*P* < 0.0001) over all histological categories for samples of HIV‐negative women and men combined. Between consecutive histological categories, a trend towards increasing methylation levels with increasing severity of disease was observed. All markers demonstrated a significant increase in methylation levels from AIN1 to AIN2 and marker *ASCL1* also for AIN3 to SCC (Fig. 1). For the other consecutive grades, significance was not reached.

### 3.3 Methylation levels in relation to HPV status

In general, we observed no significant difference between methylation levels and HPV status (HPV‐negative, lrHPV‐positive, non‐HPV16 hrHPV types, or HPV16) within histological categories of samples of HIV‐negative women and men combined (Fig. S1). Only in AIN2 samples, methylation levels of *ASCL1*, *SST* and *ZIC1* were significantly higher in HPV16‐positive samples compared with samples positive for a non‐HPV16 hrHPV type (*P* < 0.05). However, numbers for several categories were low.

### 3.4 Methylation levels in HIV‐negative women and men compared with HIV+ MSM

**Fig. 2:**
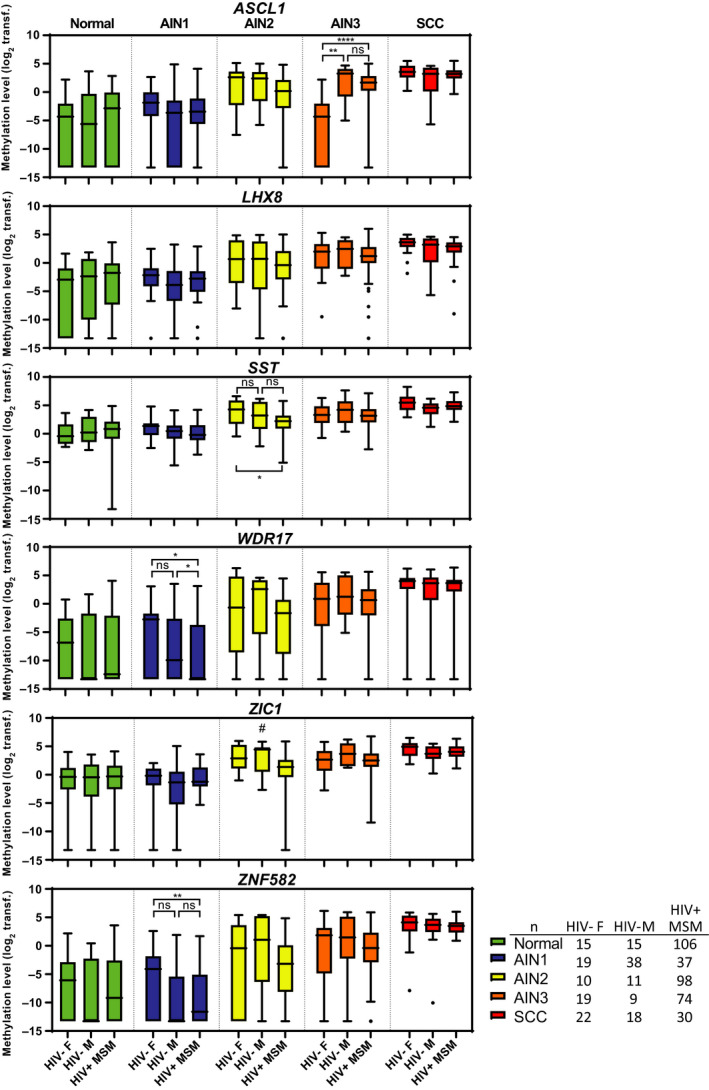
Methylation levels across samples of HIV‐negative women, HIV‐negative men and HIV+ MSM. Boxplots of DNA methylation levels relative to a reference gene *ACTB* (log_2_‐transformed ΔΔCq ratios; *y*‐axis) in the different histological categories of anal tissue samples of patient groups (*x*‐axis) for six markers: *ASCL1*, *LHX8*, *SST*, *WDR17*, *ZIC1* and *ZNF582*. The box of the boxplots bounds the IQR divided by the median, Tukey‐style whiskers extend to a maximum of 1.5 IQR beyond the box, and ● marks an outlier sample. Differences between groups per histological categories are only visualised upon a significant Kruskal–Wallis omnibus test result and followed by *post hoc* testing using the Mann–Whitney *U*‐test and Bonferroni multiple testing correction. *P < 0.05, **P < 0.01, ****P < 0.0001, ns, nonsignificant. ^#^ *P* < 0.05 on Kruskal–Wallis test but individual comparison using the pairwise Mann–Whitney *U*‐tests with the Bonferroni correction was nonsignificant. AIN: anal intraepithelial neoplasia (grades 1–3); SCC: anal squamous cell carcinoma; F: female; HIV−: HIV‐negative; M: male; ; Normal: normal control samples; transf.: transformed.

Next, we compared methylation levels with previously obtained methylation results in HIV+ MSM [11]. Figure 2 shows methylation levels of all six markers per histological category of samples of HIV‐negative women, HIV‐negative men and HIV+ MSM separately. We noted only a few significant differences (*P* < 0.05) for *ASCL1, SST*, *WDR17* and *ZNF582*, but the direction of differences between groups was not consistent. Overall, all markers show highly similar methylation levels across the different patient groups, including a trend towards increasing methylation levels with increasing severity of anal disease. Hence, in general, methylation level patterns were comparable in samples of HIV‐negative women, HIV‐negative men and HIV+ MSM.

### 3.5 Diagnostic performance of the individual methylation markers

**Fig. 3:**
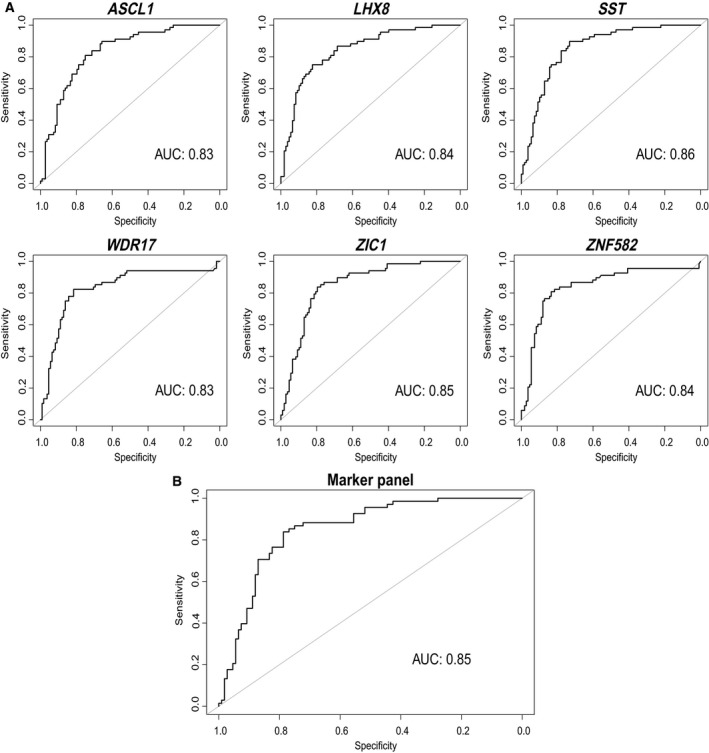
Diagnostic performance visualised with ROC curves of the ordinal regression analysis for leave‐one‐out‐cross‐validated AIN3+ detection: (A) univariable regression for the six individual methylation markers (*ASCL1*, *LHX8*, *SST*, *WDR17*, *ZIC1* and *ZNF582*) and (B) multivariable regression for the optimal marker panel (*ASCL1* and *ZIC1*). Outcome: AIN3+ (AIN3 and anal SCC).

Using ordinal regression analysis, we assessed the diagnostic performance of the six markers for the detection of AIN3+ [i.e. their ability to distinguish cases: AIN3+ (AIN3 and anal SCC; *n* = 68) from controls: ≤ AIN1 (normal control samples and AIN1; *n* = 87)]. To allow for sufficient power, we combined methylation results of HIV‐negative women and men for these analyses. All markers showed a significant distinction between cases and controls (*P* < 0.001). Following LOOCV AUCs ranged from 0.83 to 0.86 (Table 3; Fig. 3A), with the highest AUC achieved by *SST* (AUC = 0.86), followed by *ZIC1* (AUC = 0.85), *LHX8* and *ZNF582* (AUC = 0.84). *SST* classified 98% (39/40) of cancers as methylation‐positive at the *J*‐threshold (≥ 0.332), corresponding to an AIN3+ sensitivity and specificity of 90% and 73%, respectively. Using this threshold, *SST* classified 79% (22/28) of AIN3, 67% (14/21) of AIN2, 11% (6/57) of AIN1 and 30% (9/30) of normal control samples as methylation‐positive (Table 3).

**Table 3:** Ordinal regression analysis on diagnostic performance for AIN3+ detection – univariable regression of the six individual markers (*ASCL1, LHX8*, *SST*, *WDR17, ZIC1* and *ZNF582*) and multivariable regression for optimal marker panel (*ZIC1 and ASCL1*) and methylation‐positive detection rate at *J*‐threshold. LOOCV (and non‐CV for the marker panel) AUCs are reported, together with sensitivity, specificity and detection rate are for the *J*‐threshold. Outcome: AIN3+ (AIN3 and anal SCC) in anal tissue samples of (HIV‐negative) women and men.

	*ASCL1*	*LHX8*	*SST*	*WDR17*	*ZIC1*	*ZNF582*	Marker panel (non‐CV)	Marker panel (LOOCV)
AUC	0.83	0.84	0.86	0.83	0.85	0.84	0.86 (95% Cl: 0.80–0.91)	0.85
Sensitivity	81%	75%	90%	82%	84%	81%	85%	84%
Specificity	75%	82%	73%	81%	80%	83%	78%	79%
*J*‐threshold	0.516	0.574	0.332	0.544	0.469	0.560	0.483	0.525
Detection rate								
Normal	3/30 (10%)	2/30 (7%)	9/30 (30%)	3/30 (10%)	5/30 (17%)	2/30 (7%)	4/30 (13%)	3/30 (10%)
AIN1	10/57 (18%)	7/57 (13%)	6/57 (11%)	5/57 (9%)	4/57 (7%)	5/57 (9%)	7/57 (12%)	7/57 (12%)
AIN2	14/21 (67%)	10/21 (48%)	14/21 (67%)	12/21 (57%)	13/21 (62%)	11/21 (52%)	13/21 (62%)	13/21 (62%)
AIN3	18/28 (64%)	18/28 (64%)	22/28 (79%)	18/28 (64%)	19/28 (68%)	18/28 (64%)	19/28 (68%)	18/28 (64%)
SCC	37/40 (93%)	33/40 (83%)	39/40 (98%)	38/40 (95%)	37/40 (93%)	37/40 (93%)	39/40 (98%)	39/40 (98%)

### 3.6 Determination and diagnostic performance of an optimal marker panel for HIV‐negative women and men

Multivariable regression analysis was used to determine and evaluate an optimal marker panel for AIN3+ detection in HIV‐negative women and men combined. This analysis with backward selection yielded a model consisting of markers *ASCL1* and *ZIC1* as optimal panel with a non‐CV AUC of 0.86 [95% confidence interval (CI): 0.80–0.91; Table 3]. Using the *J*‐threshold (≥ 0.483), this panel provided a sensitivity of 85% and specificity of 78% and classified 98% (39/40) of the cancers, 68% (19/28) of AIN3, 62% (13/21) of AIN2, 12% (7/57) of AIN1 and 13% (4/30) of normal control samples as methylation‐positive (Table 3).

Diagnostic performance for this panel was similar after LOOCV with an AUC of 0.85 and a sensitivity of 84% and specificity of 79% at the *J*‐threshold (≥ 0.525). At this threshold, 98% (39/40) of the cancers were classified as methylation‐positive, 64% (18/28) of AIN3, 62% (13/21) of AIN2, 12% (7/57) of AIN1 and 10% (3/30) of normal control samples (Table 3).

Age and gender did not significantly contribute to the model (data not shown), supporting the combined analysis of HIV‐negative women and men. Marker *WDR17* was the only marker that classified the SCC that was missed by the panel as methylation‐positive. All hrHPV‐negative SCC were classified as methylation‐positive using the panel.

### 3.7 Application of the HIV+ MSM marker panel for AIN3+ detection in HIV‐negative women and men

By applying the marker panel (*ZNF582, ASCL1* and *SST*) previously designed for AIN3+ detection in HIV+ MSM at the non‐CV *J*‐threshold,[11] we found that this panel classified a similar proportion of samples of HIV‐negative women and men as methylation‐positive: 98% (39/40) of the cancers, 64% (18/28) of AIN3, 62% (13/21) of AIN2, 11% (6/57) of AIN1 and 17% (5/30) of normal control samples.

## 4 Discussion

The most important outcome of this study is the confirmation that akin HIV+ MSM, host cell DNA methylation also plays a role in anal carcinogenesis in HIV‐negative women and men. We observed comparable methylation level patterns in samples of HIV‐negative women and men as to our previous studies in HIV+ MSM including a significant increase in methylation levels with increasing severity of anal disease [10, 11]. All six methylation markers showed a good cross‐validated diagnostic performance for AIN3 and anal cancer detection (AUC = 0.83–0.86) in this series. Of these markers, *SST* achieved the best diagnostic performance (AUC = 0.86) for the detection of AIN3 and anal cancer with an optimal sensitivity and specificity of 90% and 73%, respectively, and classified 98% of the cancers as methylation‐positive. By evaluating combinations of markers, we composed an optimal marker panel for HIV‐negative women and men consisting of *ASCL1* and *ZIC1* with a cross‐validated AUC of 0.85. Although the marker panel had a slightly lower AUC than *SST* alone, it detected a similar proportion of SCC at a higher specificity (79%).

For HIV+ MSM, we recently showed that high methylation levels are associated with progression towards cancer. Accordingly, methylation markers may be used to stratify HGAIN at risk of progression to cancer and therefore requiring treatment, while preventing overtreatment of HGAIN lesions with a low progression risk [11]. Interestingly, the AIN and cancer detection rates obtained by present marker panel developed for HIV‐negative patients were comparable to the detection rates obtained by applying the optimal marker panel previously developed for HIV+ MSM [11], which uses the same methylation markers and qMSP assays. Therefore, we believe that current findings in samples of HIV‐negative women and men open up a broader application of methylation markers in anal cancer prevention strategies for risk groups regardless of sex or HIV status. Also for cervical (pre)cancer, comparable methylation levels and diagnostic performance of methylation analysis have been reported for HIV+ and HIV‐negative women [13, 22, 23, 24, 25]. Further validation studies in different risk populations, having different anal cancer risks and prevalence rates of HGAIN are now warranted.

We observed a comparable HPV distribution in this series of HIV‐negative women and men to what was reported in a recent meta‐analysis by Lin *et al*. [26], including an increasing trend of HPV16 positivity towards anal cancer. This trend is known to be less prominent in HIV+ MSM, in whom HGAIN lesions and SCC are more often caused by hrHPV types other than HPV16, as we also observed in our previous studies on HIV+ MSM [10, 11]. Compared to HIV+ MSM, we observed less multiple infections in our HIV‐negative series, as was also reported by Lin *et al*. [26]. The overall proportion of SCC being HPV‐positive (83%) is slightly lower to what has been reported for HIV‐negative patients (89–90%) in the meta‐analysis, although within the range of the included studies [26]. Potential explanations for the varying HPV positivity rates are differences in populations with different anal cancer aetiology, geographical differences and/or use of different HPV detection methods.

To the best of our knowledge, we are the first to have evaluated host cell DNA methylation markers in anal tissue samples from multiple target risk populations of sufficient size. Previous studies on host cell DNA methylation investigated samples of small mixed populations of predominantly HIV+ men, not allowing comparison of methylation levels between risk groups [27, 28].

Our study has several limitations. First, for this study we included samples from a HIV‐negative population consisting of patients being referred for anal complaints and patients in screening for having an increased risk of developing anal cancer. Therefore, our findings cannot be extrapolated to a screening population, but are merely a proof of principle to show the potential of methylation markers to, upon discovery with HRA, identify HGAIN biopsies in need of treatment to prevent overtreatment in a HIV‐negative referral population. Notwithstanding, present findings also indicate that methylation analysis on less‐invasive anal swabs to triage for HRA, especially in risk groups other than HIV+ MSM, who have a lower cancer risk and are not routinely screened with HRA may warrant further investigation. Second, we compared current series with our previous study on a HIV+ MSM screening population with a different study population composition, possibly leading to selection bias. HGAIN lesions found in screening programmes might be detected earlier compared with lesions found in a referral population and could therefore represent lesions earlier during carcinogenesis. Methylation levels in the present study were comparable to those in our previous study on samples of a HIV+ MSM screening population. Nevertheless, the different study populations may mask a potential difference in cancer risk between HIV+ and HIV‐negative patients. This difference in populations, together with different multivariable models for marker panel development, hindered a direct comparison of the marker panels developed for HIV‐negative patients and HIV+ MSM. Third, although medical records were thoroughly reviewed, risk factors and HIV status were not always documented or available. HIV‐negativity could therefore not be confirmed for some of the women with anal cancer. Fourth, while maximising our series by including all available archival tissue samples, included samples were not balanced across the histological categories. Also, occasionally multiple samples per patient were included. As a result, the independent assumption between samples is violated since observations from the same patient tend to be more correlated. To account for this correlation structure within the same patients, we incorporated a random subject effect in our model to minimise sampling bias. Last, although this is the largest study evaluating host cell DNA methylation markers in HIV‐negative women and men to date, numbers were still generally too low for several subgroups analyses, for example comparing HIV‐negative women and men. Although we found no significant contribution of gender in the multivariable analysis and we could combine HIV‐negative women and men, we acknowledge that this was still a heterogeneous group with different risk factors. Moreover, by comparing results with our previous series on HIV+ MSM, which was much larger, comparisons could have been influenced by an imbalance in numbers.

## 5 Conclusions

We showed that similar to anal carcinogenesis in HIV+ MSM, host cell DNA methylation also plays a role in anal carcinogenesis in HIV‐negative women and men. The high resemblance in methylation levels and good diagnostic performance of the methylation markers in samples of both HIV‐negative and HIV+ patients open up a broad application of methylation markers in anal cancer prevention strategies for all risk groups of anal cancer.

## Conflict of interest

RDMS and DAMH are minority stockholders of Self‐screen BV, a spin‐off company of VUmc, which owns patents on methylation markers and HPV detection. DAMH has been on the speaker's bureau of Qiagen and serves occasionally on the scientific advisory boards of Pfizer and Bristol‐Myers Squibb. HJCdV received financial compensation or goods for research from MediGene, Gilead and MSD; financial compensation for presentations from Abbott and Janssen; and financial compensation for advice to MediGene and Novartis. All other authors report no potential conflicts.

### Peer Review

The peer review history for this article is available at https://publons.com/publon/10.1002/1878‐0261.12926.

## Author contributions

RDMS, JMP and HJCdV are principal investigators of the study. RPvdZ and RDMS designed the study. RPvdZ, CJMvN, DAMH, HJCdV, JMP and RDMS were involved in providing and collecting clinical material and data collection. RPvdZ and TJtB performed the laboratory work. RPvdZ and RDMS performed the data analysis. RPvdZ and IM performed the statistical analysis. RPvdZ managed the database. RPvdZ and RDMS drafted the manuscript. All authors critically reviewed the manuscript and approved the final version. All authors had full access to all of the data in the study and can take responsibility for the integrity of the data and the accuracy of the data analysis and believe that the manuscript represents honest work. RDMS affirms that the manuscript is an honest, accurate and transparent account of the study being reported, that no important aspects of the study have been omitted and that any discrepancies from the study as planned have been explained; and has full responsibility for the decision to submit for publication.

## Supporting information


**Fig. S1.** Boxplots of DNA methylation levels relative to a reference gene ACTB (log2‐transformed ΔΔCq ratios; y‐axis) in relation to HPV status in the different histological categories of anal tissue samples of HIV‐negative women and men combined (x‐axis) for six markers.

**Table S1**. Number of included tissue samples of HIV‐negative men (A) en HIV‐negative women (B), including age at biopsy, anatomical location, HIV status and risk factors per histological category.

## Data Availability

Study data that underlie the results reported in this article (text, tables, figures and appendices) and a data dictionary will be available for 5 years after publication after deidentification and at request to the corresponding author. Data will be securely transferred to researchers, who provide a methodologically sound research proposal and only to achieve aims in the approved proposal, after: approval of the ethics review board; additional approval of study participants (if applicable); and signing a data access agreement.
