# Supplementary material for: DNA methylation markers have universal prognostic value for anal cancer risk in HIV‐negative and HIV‐positive individuals
Source: Mol Oncol. 2021 Mar 16;15(11):3024–36. doi: 10.1002/1878-0261.12926 (PMC8564631; doi:10.1002/1878-0261.12926)
Supplement: Supplementary file 1 — Fig. S1. Boxplots of DNA methylation levels relative to a reference gene ACTB (log2‐transformed ΔΔCq ratios; y‐axis) in relation to HPV status in the different histological categories of anal tissue samples of HIV‐negative women and men combined (x‐axis) for six markers. [file MOL2-15-3024-s001.pdf]

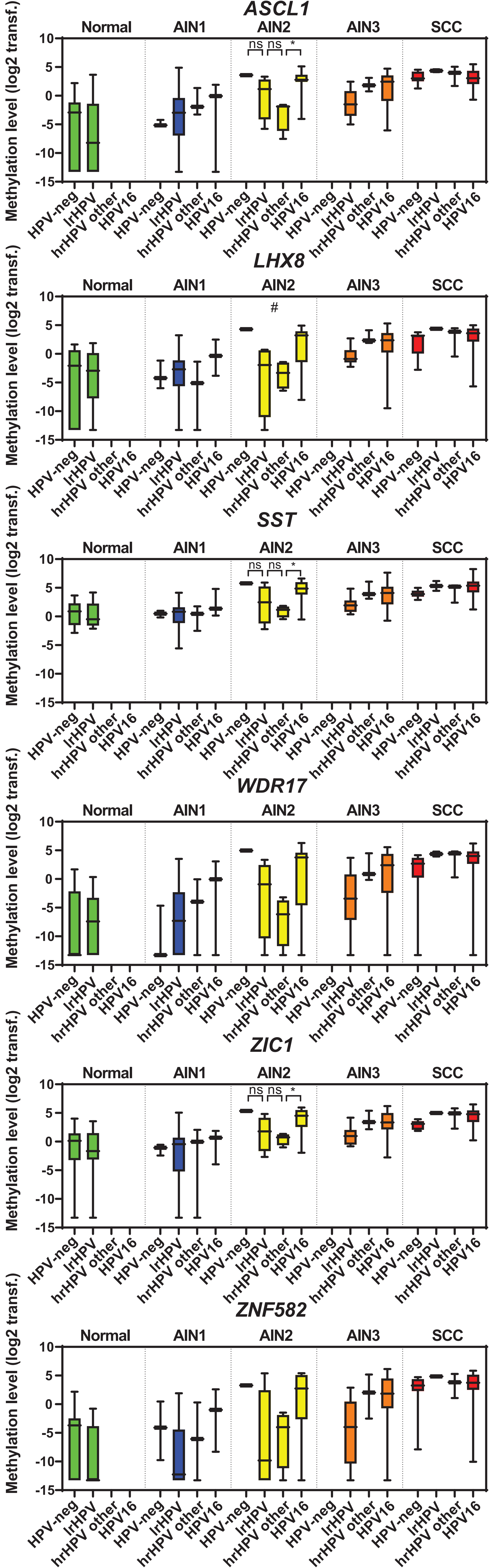

Supplementary figure 1 (Fig. S1) Methylation levels in relation to HPV status. Boxplots of DNA methylation levels relative to a reference gene *ACTB* (log2-transformed ΔΔCq ratios; *y*-axis) in relation to HPV status in the different histological categories of anal tissue samples of HIV-negative women and men combined (*x*-axis) for six markers: *ASCL1, LHX8, SST, WDR17, ZIC1* and *ZNF582*. HPV status: positive for ≥1 HPV type in the WTS belonging to hierarchical (from low to high) risk classes (HPV-negative, lrHPV-positive, hrHPV other (non-HPV16 hrHPV-positive), HPV16-positive).[29, 30] The box of the boxplots bounds the interquartile range (IQR) divided by the median and Tukey-style whiskers extend to a maximum of 1.5 IQR beyond the box. Differences between HPV status groups per histological categories are only visualised upon a significant Kruskal-Wallis omnibus test result and followed by *post hoc* testing using the Mann-Whitney *U*-test and Bonferroni multiple testing correction: * $P < 0.05$, ns: nonsignificant. # $P < 0.05$ on Kruskal-Wallis test but individual comparison using pairwise Mann-Whitney *U*-tests with Bonferroni correction were nonsignificant. Normal, normal control samples; AIN, anal intraepithelial neoplasia (grades 1-3); HPV, human papillomavirus; HPV-neg, HPV-negative; hrHPV, high-risk HPV; lrHPV, low-risk HPV; SCC, squamous cell carcinoma; transf., transformed; WTS, whole tissue section.

n	HPV-neg	lrHPV	hrHPV other	HPV 16
Normal	17	13	0	0
AIN1	3	48	3	3
AIN2	1	4	4	12
AIN3	0	6	3	19
SCC	7	2	3	28
