# Supplementary material for: DNA methylation markers have universal prognostic value for anal cancer risk in HIV‐negative and HIV‐positive individuals
Source: Mol Oncol. 2021 Mar 16;15(11):3024–36. doi: 10.1002/1878-0261.12926 (PMC8564631; doi:10.1002/1878-0261.12926)
Supplement: Supplementary file 2 — Table S1. Number of included tissue samples of HIV‐negative men (A) en HIV‐negative women (B), including age at biopsy, anatomical location, HIV status and risk factors per histological category. [file MOL2-15-3024-s002.pdf]

Supplementary table 1. Number of included tissue samples of HIV-negative men (A) en HIV-negative women (B), including age at biopsy, anatomical location, HIV status, and risk factors per histological category

A.

			**Anatomical location**			**Risk factors (HIV-negative men)**						
						**HIV**						
**Histological category**	**Subtotal**	**Age at biopsy, years**	**Anal canal**	**Peri-anal**	**Not provided**	**neg**	**presumed neg**	**MSM**	**SOTR**	**Other systemic immunosupp.**	**IBD**	**Not reported/ no risk factor**
Normal	15	53 [46-69]	12	3	0	15	0	10	0	2	0	3
AIN1	38	44.5 [29-54]	36	2	0	38	0	22	2	3	0	11
AIN2	11	52 [45-60]	10	1	0	11	0	8	0	1	0	2
AIN3	9	63 [52-68]	8	1	0	9	0	5	0	1	0	3
SCC	18	56.5 [51-65]	5	10	3	18	0	1	2	1	1	13
**Total**	**91**	**52 [41-62]**	**71**	**17**	**3**	**91**	**0**	**46**	**4**	**8**	**1**	**32**

B.

			**Anatomical location**			**Risk factors (HIV-negative women)**											
						**HIV**											
**Histological category**	**Subtotal**	**Age at biopsy, years**	**Anal canal**	**Peri-anal**	**Not provided**	**neg**	**presumed neg**	**cervical (pre-) cancer**	**vulvar (pre-) cancer**	**vulvar + cervical (pre-)cancer**	**SOTR**	**SOTR + cervical/ vulvar (pre-) cancer**	**Other systemic immunosupp.**	**cervical (pre-) cancer + other systemic immunosupp.**	**IBD**	**IBD + cervical (pre-) cancer**	**Not reported/ no risk factor**
Normal	15	61 [46-67]	12	3	0	15	0	4	2	2	0	0	0	1	1	1	4
AIN1	19	49 [41-57]	16	3	0	19	0	4	1	4	0	2	2	0	1	1	4
AIN2	10	44 [39-58]	5	5	0	10	0	2	3	2	0	1	1	0	0	0	1
AIN3	19	52 [4-62]	9	10	0	19	0	2	6	4	0	2	0	1	1	0	3
SCC	22	58.5 [44-77]	7	12	3	4	18	0	3	2	0	0	0	0	1	0	16
**Total**	**85**	**52 [44-65]**	**49**	**33**	**3**	**67**	**18**	**12**	**15**	**14**	**0**	**5**	**3**	**2**	**4**	**2**	**28**

Data are n, numbers or median [IQR]. Numbers represent samples. Consequently, patients with multiple sample can be represented in multiple categories. Abbreviations: Normal: normal control samples; AIN: anal intraepithelial neoplasia (grades 1-3); HIV-neg: HIV-negative; IBD: Inflammatory Bowel Disease (Crohn's disease or ulcerative colitis); immunosupp.: immunosuppressants; SCC: anal squamous cell carcinoma; SOTR: solid organ transplantation recipient.
